# Tartar Emetic in Obstetric Practice

**Published:** 1836

**Authors:** 


### Art. V.—Tartar Emetic in Obstetric Practice.

In our excellent cotemporary, the American Journal of the Medical Sciences, we have a valuable paper on the use of tartar emetic in obstetric practice, by Dr. Kennedy of the Dublin Lying-in Hospital. The writer informs us that his remarks are the result of many years experience, in an ample field of obstetric observation. The opportunities afforded the managers of large hospitals in Europe give them many advantages over our practitioners in testing the value of different medicines in various diseases. Dr. Kennedy is an indefatigable student of nature, as well as a scientific man, and hence his remarks may be relied on as strictly correct' We have for some time been in the habit of prescribing tartar emetic, in small doses, in cases of protracted labor from a rigid state of the os uterus, where venesection could not be resorted to. We believe that such cases occur more frequently in European cities, at least in large hospitals, than they do in our own country, for here bloodletting is not only admissible, but required in nineteen cases out of twenty, especially in a country practice where the females are generally of a strong plethoric habit. They partake but little of the phlegmatic temperament, so often present in the inmates of lying-in hospitals, where tartar emetic as directed by Dr. Kennedy would be essentially useful. In many instances the medicine is beneficial after bloodletting. We take the following remarks, on tedious labor from rigidity of the os uteri and vagina, from the paper referred to.

"In tedious labor, from rigidity of the uterus, the os is found slightly gaping with a thickened, tense state of the lips, and usually much heat of the parts. Bleeding from the arm, and on the continent the use of the warm bath have been had recourse to in these cases. Bleeding is attended with marked benefit when there is a full bounding pulse, in a strong plethoric habit: but, as a general practice, it is not unattended with inconveniences often of a very serious nature. It certainly procures relaxation of the os uteri, but along with this it causes depression of too permanent a nature, and may thus seriously interfere with the future progress of the labor. Tartar emetic solution has been successfully employed in producing relaxation of the os uteri in these cases, and possesses the advantage of being much less permanently debilitating. It is an agent by which the system can be with safety brought into a much greater degree of temporary depression: between which state and relaxation of the contractile tissues, a marked connexion holds, if they do not absolutely stand in the relation of cause and effect. The principal recommendation, however, to tartar emetic in these cases is, that in its use, the power of regulating the necessary degree of lowering the system, exists completely in the hands of the practitioner, as he has only to increase, or diminish, or suspend the dose, in order to produce the effect he wishes; and, when the necessary effect is produced, the withdrawal of the medicine leaves the vital energies but little impaired. The medicine has been used in the ordinary nauseating doses, as in pneumonia, 5 or 6 grains of the tartrate of antimony, dissolved in eight ounces of water, and generally 20 drops of laudanum, and a small quantity of syrup added; one, two, or more table-spoonsful of this mixture are given at intervals of from fifteen minutes to two, three, or four hours, according to the effect it produces, and the necessity that exists for bringing the patient speedily or otherwise under its influence. Sometimes it is necessary to cause free vomiting in the first instance, or the ordinary doses produce no nauseating effect; in such cases the laudanum is better withheld, but may be added afterwards if necessary. In other cases the medicine acts too violently as an emetic or produces purging; here increasing the quantity of the laudanum, and diminishingthe dose, or allowing a longer interval to intervene between the doses, will be necessary. The accoucher must, therefore, watch carefully the effects of the medicine during its administration in every case in which it is employed; these observations applying with equal force to the forms of disease in which its utility has been proved. Under some of the circumstances described, or where the antimonial in every dose and form disagreed with the patient, small and frequently repeated doses of hippo [ipecac] have been substituted (three to five grains every hour or second hour,) and with good effect, not only in rigidity of the uterus, but in the other diseases in which tartar emetic was found efficacious. It should be mentioned, that neither tartar emetic nor venesection have been relied upon singly in some cases where it has been necessary to produce a speedy dilatation of the os uteri, and where the plethoric state of the system described was present. In such, after depletion, the patient was kept for some hours under the influence of the nauseating mixture. One case, in particular, of a most threatening nature, may be mentioned, in which a strong robust woman was brought into hospital with the arm forced into the vagina, through a tense, rigid, and slightly dilated os uteri. She was so treated, and with the best results. There is a somewhat different state of the os uteri, in which it occasionally dilates very tardily also; here the lip of the uterus is thin and stretched over the head of the child, not affording the sensation of heat or rigidity of fibre observed in the case above described. The extract of belladonna appeared of service in a few of these cases, although its general efficacy appeared very questionable. In two cases of rigid os uteri, in which it was freely used, its application was followed by head symptoms and depression of pulse; in one of which even insensibility and stertor were present. It was, however, tried in many other cases, without being followed by these unpleasant effects. The last described state of the os uteri is also occasionally benefitted by the nauseating medicine. It may depend, however, upon other causes, not under our present consideration, nor is it looked upon with the same anxiety by the accoucher as a cause of tedious labor. In concluding this branch of our subject, let it not be inferred from what has preceded, that tartar emetic will invariably succeed in procuring dilatation of the os uteri; as it is in some cases found quite unavailing, in others inadmissible. Its efficacy, however, in a great many cases in which it has been used, fully warrant its attracting the attention of the obstetrician, and its success will depend much on a proper selection being made of the cases in which it is available."

